# Contact Dermatitis and Medical Adhesives: A Review

**DOI:** 10.7759/cureus.14090

**Published:** 2021-03-24

**Authors:** Nicholas J Thornton, Bernard R Gibson, Andrew M Ferry

**Affiliations:** 1 Dermatology, University of Texas Medical Branch, Galveston, USA; 2 Plastic and Reconstructive Surgery, Baylor College of Medicine, Houston, USA

**Keywords:** contact dermatitis, medical adhesives, contact dermatitis and medical adhesives, mastisol, dermabond, benzoin, allergic contact dermatitis (acd), irritant contact dermatitis (icd)

## Abstract

In more recent years, the use of medical adhesives in lieu of sutures or staples has become increasingly common for the closure of post-surgical and traumatic incisions in areas of the skin where tension is low. While medical adhesives possess many advantages and little risk of adverse side effects, there are increasing numbers of accounts in the medical literature of allergic contact dermatitis (ACD) caused by specific components contained within the medical adhesives.

The goal of this paper is to provide physicians with a differential diagnosis when faced with complications after the use of medical adhesives for wound closure. Additionally, this paper aims to delineate the differences among the most commonly used adhesives, provide a rationale for assessing an individual’s personal risk of developing ACD, and to highlight the unique advantages and disadvantages of each adhesive.

Dermabond® appears to be the most versatile adhesive with the lowest risk of ACD. However, because of its high cost, it may not be appropriate for all patients. While Mastisol® can only be utilized in combination with a dressing, such as Steri-Strips®, it is much more affordable than Dermabond and is still capable of providing an effective wound closure. Due to these factors, it is our recommendation that Dermabond is considered the first-line medical adhesive due to its versatility and strength, while Mastisol can be readily employed in situations with financial consideration.

As the number of patients treated with medical adhesives continues to grow, physicians should anticipate an increase in the number of cases of ACD secondary to adhesive sensitization. It is imperative for physicians to be able to differentiate between a case of ACD and another potentially more serious complication, such as cellulitis. We hope that this paper will assist providers in distinguishing adhesive-induced ACD and other complications, identifying patients at risk of ACD from adhesive use, and provide a basis for which adhesives are most appropriate for any given patient.

## Introduction and background

Ideal wound closure methods are continually evolving as new technologies emerge. In more recent years, the use of medical adhesives in lieu of sutures or staples has become increasingly common for the closure of post-surgical and traumatic incisions in low-tension areas. Medical adhesives can be an ideal method of wound repair due to their ease of application resulting in decreased time to closure, ability to create an effective antimicrobial seal, decreased necessity for wound care follow-up, and in the correct applications, improved aesthetic outcome. 

For the majority of patients, medical adhesives are well-tolerated and preferable to methods requiring more frequent follow-up care. In a select minority of patients, however, sensitization and subsequent allergic contact dermatitis (ACD) may be observed [1-6]. 

Arguably the most feared complication following closure of any wound is infection, and it is imperative for physicians to be able to distinguish cellulitis from contact dermatitis to initiate the appropriate treatment regimen. 

This paper aims to review the most common medical adhesives to compare their effectiveness and explore the unique considerations of each. Additionally, we have constructed a simple table to assist in distinguishing ACD secondary to adhesive use and the other common dermatologic complications following laceration repair.

## Review

Methods

This review utilized the Medline database via the PubMed search engine to identify articles published from 1980 to 2020 examining medical adhesives and contact dermatitis. Keywords used were “ACD,” “ICD (irritant contact dermatitis),” “dermatitis,” “medical adhesives,” “medical adhesives and contact dermatitis,” and “cellulitis.” Over 50 articles were identified and 20 were selected for inclusion. 

Results

**Figure 1:**
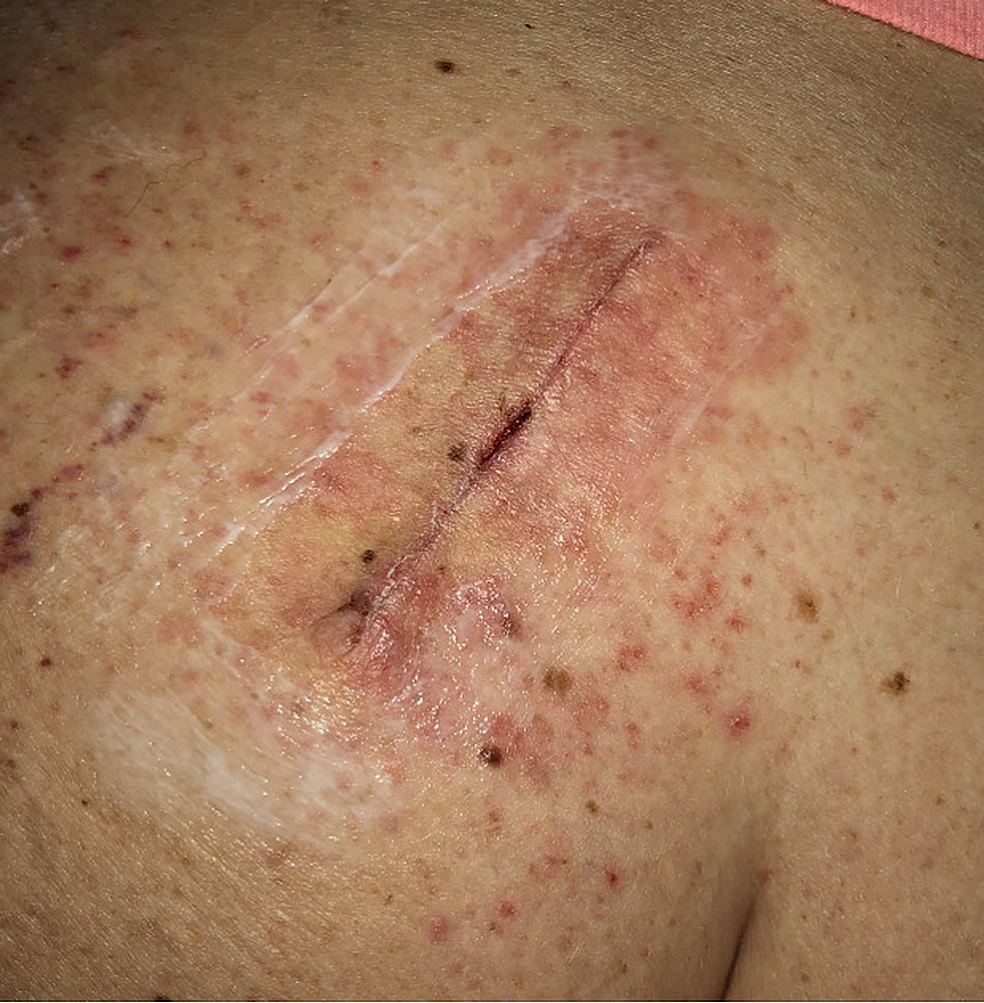
Allergic contact dermatitis following the use of Mastisol and Steri-Strips

The most commonly used adhesives are cyanoacrylates, benzoin derivatives, and Mastisol. All of these products are used similarly for achieving wound closure. They are applied topically in low-tension areas of the body instead of placing sutures or staples. In addition to the established potential complications following wound closure, the medical adhesives possess the risk of causing ACD of variable severity, such as the case seen in Figure 1.

This reaction is believed to occur via a delayed type IV hypersensitivity reaction involving T-cells, macrophages, and monocytes [2]. In this reaction, antigen-presenting cells (commonly macrophages) containing the culprit antigen bound to the cell via major histocompatibility complex II (MHC II) molecules are recognized by CD4+ type 1 T helper (Th1) cells. The antigen-presenting macrophage cells secrete interleukin (IL)-12, causing further proliferation of additional CD4+ T cells. The CD4+ T cells primarily release interferon-gamma and IL-2, which trigger the cascade of Th1 cytokines that effectively mediate this inflammatory immune response [2, 7]. 

The clinical result of this hypersensitivity reaction is local inflammation, pruritus, and, in severe cases, epidermal necrosis. The time frame in which this reaction will occur depends on whether or not the patient has experienced previous sensitization or not. Primary sensitization will result in manifestations seven to 10 days after exposure, whereas patients with a history of sensitization will experience symptoms within 12 to 48 hours [2]. Symptoms are generally worse with repeated exposures. Primary management involves diligent avoidance of the offending antigen. Symptoms can be managed with oral antihistamines and a combination of oral and topical corticosteroids [2]. 

**Figure 2:**
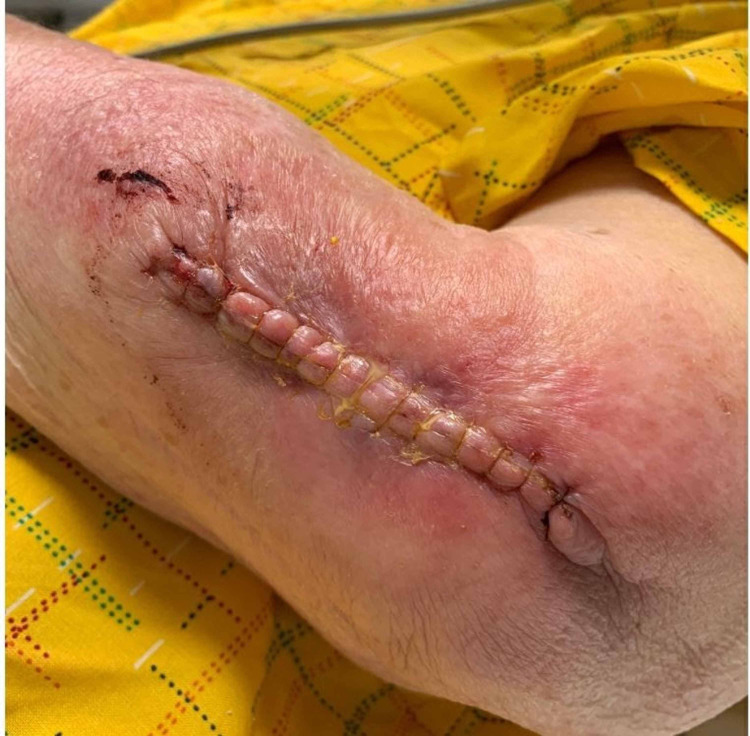
Cellulitis after wound closure with sutures

When dealing with a suspected case of ACD following the use of an adhesive, it is imperative for providers to develop a robust differential and be able to differentiate between other common complications, such as cellulitis. An example of cellulitis following wound closure can be seen in Figure 2.

A potential differential for ACD following medical adhesive usage is included in Table 1 below.

**Table 1:** Differential Diagnosis for Allergic Contact Dermatitis (ACD) Following Use of Medical Adhesives This table includes a basic differential for physicians faced with wound healing complications. The table includes the cause, clinical appearance, and treatment for each diagnosis [8-10].

Diagnosis	Cause	Clinical Appearance	Treatment
Allergic Contact Dermatitis	Delayed hypersensitivity reaction	Erythematous macules, papules, and plaques. Vesiculation and serous drainage may be observed. Lesions may appear anywhere on the body in severe systemic cases (Figure *1*).	Avoidance of allergen; steroids
Irritant Contact Dermatitis	Direct exposure to irritants	Well-defined erythematous patches or plaques distributed at sites of irritant exposure [8-9].	Avoidance of allergen; steroids
Cellulitis	Infection	Erythema, edema, warmth, and tenderness to palpation. Unilateral distribution with smooth poorly defined borders. Systemic symptoms, such as fever, chills, malaise, or leukocytosis (Figure *2*) [10].	Antimicrobial therapy directed at the offending organism(s)
Stasis Dermatitis	Chronic venous insufficiency resulting in	Bilateral, poorly defined erythema with or without hyperpigmentation, serous drainage, and desquamation. Commonly on the lower extremities [9].	Topical corticosteroids; wet compress; compression stockings
Lipodermatosclerosis	Chronic stasis dermatitis	Acute: Poorly defined erythema, edema, warmth, and pain above the medial malleolus. Chronic: Sharply demarcated indurated or sclerotic plaques generally affecting the area below the knee and above the ankle. Skin is often bronze or brown due to deposition of hemosiderin, and prominent varicosities and ulceration may be seen depending on disease course [9].	Compression stockings; treatment of underlying venous insufficiency
Lymphedema	Disruption of lymphatic system	Often unilateral localized erythema, induration, and edema of an affected extremity. Hyperkeratosis, hyper or hypopigmentation may be seen. Lack of systemic symptoms [9].	Compression; lymphatic massage
Eosinophilic Cellulitis	Recurrent hypersensitivity reaction	Localized edema, induration, and erythema with well-defined demarcated borders about the affected extremity. Chronic cases may present with firm indurated plaques [9].	Topical or oral steroids
Papular Urticaria	Dermal hypersensitivity to an insect bite	Can present as numerous intensely pruritic urticarial papules or, in severe cases, as large indurated erythematous plaques reminiscent of those seen in cellulitis. Commonly occurring 1 to 2 hours after bites occur [9].	Steroids; antihistamines

The most common adhesives are further delineated below and their unique characteristics are discussed. 

Histoacryl Blue (N-Butyl-2-Cyanoacrylate)

Histoacryl blue or n-butyl-2-cyanoacrylate was the first cyanoacrylate skin adhesive created for use as a topical wound closure in 1968 [11]. It is used to achieve wound closure by being applied in beads to the wound edge and allowed to dry with or without the use of Steri-Strips. It is ideal for incisions in low-tension areas, but due to the advent of stronger, more flexible adhesives, it is less commonly used today. 

Dermabond (2-Octyl Cyanoacrylate)

Dermabond or 2-octyl cyanoacrylate is the first topical skin adhesive to gain FDA approval and is four times stronger than Histoacryl® Blue with improved flexibility [8]. It is used alone or in combination with a self-adhesive polyester mesh. This combination system is known as the Dermabond Prineo® Skin Closure System [12]. This system is utilized in place of sutures for achieving closure of incisional or traumatic lacerations [13]. The self-adhesive mesh is laid over the wound and then coated with 2-octyl cyanoacrylate in liquid form. Shortly after exposure to air, the liquid acrylic monomers will cure into an acrylic polymer form that binds tightly with keratin [11, 14]. This reaction provides a strong, minimally invasive seal of the wound with less need for maintenance and follow-up wound care. One 2017 case report by Bitterman and Sandhu implicated humidity as being an important factor in the polymerization process [15]. This paper argued that since the majority of cases of ACD to Dermabond have occurred in arid geographical climates, the lack of moisture delayed the polymerization process. This delay allowed more time for antigen-presenting cells to become sensitized to the acrylic monomers implicated in causing ACD [16-17]. Current literature indicates that 1% - 1.4% of patients will experience some degree of allergic contact dermatitis following exposure to acrylic monomers [17]. 

Benzoin

Tincture of benzoin is another popular topical liquid adhesive composed of 10% benzoin in ethanol. Compound tincture of benzoin (CTB) is a similar preparation consisting of benzoin, styrax, balsam of Tolu, and aloe suspended in ethanol [3]. Both of these liquids are used to assist in adhering Steri-Strips to the skin surrounding the lacerations. Tincture of benzoin and CTB are utilized in the same situations as Dermabond and can effectively replace sutures or staples in traumatic or incisional lacerations. 

Although rare, some patients have been reported to develop allergic contact dermatitis following exposure to either of these benzoin tinctures. Additionally, cross-reactivity with similar compounds, such as gum mastic, colophonium (colophony), and fragrance substances, has been observed in patients with identified allergic reactions to benzoin tincture or CTB [3-4, 18]. 

Mastisol

Mastisol is a liquid adhesive consisting of ethanol, acetone, methyl salicylate, gum mastic, styrax, and water. It is used similarly to benzoin-containing tinctures and Dermabond for achieving wound closure in place of sutures or staples. 

Although also uncommon, there have been case reports describing Mastisol-related allergic contact dermatitis [4-5]. Those with an allergic response to Mastisol have the potential for cross-reactivity with similar allergens, including fragrances, balsam of Peru, colophony, and tea tree oil [19]. 

Results from a large investigational study comparing CTB and Mastisol indicate that Mastisol has significantly less cross-reactivity, less incidence of skin discoloration, and is a 7 to 8 times stronger adhesive than CTB [4]. Because of these considerations, Mastisol is considered a superior adhesive to CTB or tincture of benzoin in patients with or without suspected allergic reactivity [4]. 

A summary of the adhesives described is included in Table 2 below.

**Table 2:** Commonly Used Medical Adhesives This table depicts the most commonly utilized medical adhesives. The table includes the adhesive’s common name, ingredients, cost per mL, and the possibility for sensitization. Additionally, compounds known to cross-react with each adhesive are listed, as well as unique considerations for each adhesive.

Adhesive	Active Ingredients	Cost	Sensitization Possible	Common Cross-Reactivity	Special Considerations
Histoacryl Blue	N-butyl-2-cyanoacrylate	~$44 per mL	yes	Acrylates; acetylates; formaldehyde	Inferior strength compared to Dermabond; rarely in use today [11]
Dermabond	2-octyl cyanoacrylate	~$130 per mL	yes	Acrylates; acetylates; formaldehyde	Most expensive adhesive with similar effectiveness as Mastisol; decreased cross-reactivity [8, 11, 13]
Benzoin	Benzoin 10%; ethanol	~$0.26 per mL	yes	Balsam of Peru; styrax; eugenol; vanilla; α‐pinene; benzyl alcohol; benzyl cinnamate	Considerably inferior adhesive qualities [3-4, 18]
Compound Tincture of Benzoin	Benzoin 10%; styrax 8%; balsam of Peru 4%; aloe 2%	~$0.20 per mL	yes	Balsam of Peru; styrax; eugenol; vanilla; α‐pinene; benzyl alcohol; benzyl cinnamate; colophonium	Considerably inferior adhesive qualities with increased risk of cross-reactivity [3-4, 18]
Mastisol	Acetone; methyl salicylate; gum mastic; styrax; water	~$72 per mL	yes	Gum mastic; Majantol®; balsam of Peru; hydroperoxides of linalool; gum styrax	Superior adhesive strength to benzoin products; less risk of cross-reactivity; decreased cost compared to Dermabond [4-5, 19]

Discussion

Intuitively, as the use of medical adhesives for wound closure continues to increase, greater numbers of patients will develop sensitization and subsequent ACD to these topical compounds. It is paramount for providers to be able to distinguish these cases of ACD from cellulitis and other common adverse events as their treatments vary considerably [16]. Arguably, the most effective tool used in differentiating ACD from other complications is thorough history-taking. Additionally, while the clinical appearance of cellulitis may be quite similar to ACD and other mimics, the presence of substantial tenderness to palpation, in addition to systemic signs of fever or leukocytosis, should always raise suspicion for cellulitis [9]. 

When selecting a topical adhesive to use for wound closure, it should be noted that they each possess unique qualities that must be considered for each patient. While 2-octyl cyanoacrylate has been shown to possess the strongest adhesive qualities, it is, by far, the most expensive and possesses the risk of ACD in patients with a history of allergy to acetylate or aldehyde-containing substances [1]. Benzoin-containing products are by far the cheapest medical adhesives; however, they possess inferior strength and significantly higher rates of allergic cross-reactivity with other similar compounds (especially with the use of CTB) [3-4]. While Mastisol is considerably more expensive than benzoin products, it is offered at roughly half the cost of Dermabond. Mastisol is considerably stronger than benzoin products with significantly lower rates of allergic cross-reactivity [4]. While Dermabond can be utilized with or without the application of adhesive mesh dressings, such as Steri-Strips, the other adhesives are only used in combination with Steri-Strip-like dressings. 

Due to the unique advantages and disadvantages of each medical adhesive, the specific considerations of each patient must be respected in order to select the most appropriate adhesive. Dermabond appears to be the most versatile adhesive with the lowest risk of ACD; however, because of its high cost, it may not be appropriate for all patients. While Mastisol can only be utilized in combination with a dressing, such as Steri-Strips, it is much more affordable than Dermabond and is still capable of providing an effective wound closure. Due to these factors, it is our recommendation that Dermabond be considered the first-line medical adhesive due to its versatility and strength, while Mastisol can be readily employed in situations with financial consideration. Benzoin products, while inexpensive, possess inferior adhesive properties with a significantly higher risk of ACD and allergic cross-reactivity. For these reasons, we believe benzoin products should only be utilized if other adhesives are unavailable or unaffordable to the patient. 

In patients with a prior history of ACD to adhesives or allergy to substances known to possess allergic cross-reactivity with the adhesives, it may be appropriate to utilize sutures or staples for wound closure to alleviate the risk of ACD. It may be beneficial to diagnose patients with suspected ACD by utilizing patch testing [9]. A thorough history, however, should always take precedence when attempting to elucidate the root cause of a wound reaction. 

Future studies may wish to examine the benefit of screening all patients for adhesive allergy or allergy to compounds known to cross-react with medical adhesives when selecting wound closure methods. As new methods of wound closure continue to be developed, they will need to be assessed for their potential to cause allergic reactions [20]. 

## Conclusions

Based on the results of our review, it is our recommendation that Dermabond be considered the first-line and superior medical adhesive, with the high cost being the primary disadvantage of this product. In patients with financial concerns and no history of ACD to Mastisol or its individual components, we recommend Mastisol as the second-line medical adhesive. Physicians should consider ACD in their differential when faced with complications of wound healing following the use of medical adhesives and understand the key features, allowing it to be differentiated from cellulitis or other mimics.

## References

[1] Davis MD, Stuart MJ (2016). Severe allergic contact dermatitis to Dermabond Prineo, a topical skin adhesive of 2-octyl cyanoacrylate increasingly used in surgeries to close wounds. Dermatitis.

[2] Kostner L, Anzengruber F, Guillod C, Recher M, Schmid-Grendelmeier P, Navarini AA (2017). Allergic contact dermatitis. Immunol Allergy Clin North Am.

[3] Sasseville D, Saber M, Lessard L (2009). Allergic contact dermatitis from tincture of benzoin with multiple concomitant reactions. Contact Dermatitis.

[4] Lesesne CB (1992). The postoperative use of wound adhesives. Gum mastic versus benzoin, USP. J Dermatol Surg Oncol.

[5] Shaw DW (2020). Contact dermatitis from gum mastic (Pistacia lentiscus) and gum storax (Liquidambar styraciflua) in Mastisol-allergic patients. Dermatitis.

[6] Hivnor CM, Hudkins ML (2008). Allergic contact dermatitis after postsurgical repair with 2-octylcyanoacrylate. Arch Dermatol.

[7] Mowad CM, Anderson B, Scheinman P, Pootongkam S, Nedorost S, Brod B (2016). Allergic contact dermatitis: patient management and education. J Am Acad Dermatol.

[8] Bains SN, Nash P, Fonacier L (2019). Irritant contact dermatitis. Clin Rev Allergy Immunol.

[9] Keller EC, Tomecki KJ, Alraies MC (2012). Distinguishing cellulitis from its mimics. Cleve Clin J Med.

[10] Bailey E, Kroshinsky D (2011). Cellulitis: diagnosis and management. Dermatol Ther.

[11] García Cerdá D, Ballester AM, Aliena-Valero A, Carabén-Redaño A, Lloris JM (2015). Use of cyanoacrylate adhesives in general surgery. Surg Today.

[12] Dunst KM, Auboeck J, Zahel B, Raffier B, Huemer GM (2010). Extensive allergic reaction to a new wound closure device (Prineo). Allergy.

[13] Knackstedt RW, Dixon JA, O'Neill PJ, Herrera FA (2015). Rash with Dermabond Prineo skin closure system use in bilateral reduction mammoplasty: a case series. Case Rep Med.

[14] Tomb RR, Lepoittevin JP, Durepaire F, Grosshans E (1993). Ectopic contact dermatitis from ethyl cyanoacrylate instant adhesives. Contact Dermatitis.

[15] Bitterman A, Sandhu K (2017). Allergic contact dermatitis to 2-octyl cyanoacrylate after surgical repair: humidity as a potential factor. JAAD Case Rep.

[16] Worsnop F, Affleck A, Varma S, English J (2007). Allergic contact dermatitis from Mastisol mistaken for cellulitis. Contact Dermatitis.

[17] Drucker AM, Pratt MD (2011). Acrylate contact allergy: patient characteristics and evaluation of screening allergens. Dermatitis.

[18] Scardamaglia L, Nixon R, Fewings J (2003). Compound tincture of benzoin: a common contact allergen?. Australas J Dermatol.

[19] Hood CR Jr, Cornell RS, Greenfield B (2016). Liquid adhesive contact dermatitis after bunionectomy: a case report and literature review. J Foot Ankle Surg.

[20] Mattick A (2002). Use of tissue adhesives in the management of paediatric lacerations. Emerg Med J.

